# Epidemiological and virological characteristics of people living with HIV on antiretroviral treatment for more than 6 months in virological failure in Brazzaville, Republic of Congo

**DOI:** 10.1099/acmi.0.000805.v3

**Published:** 2024-11-11

**Authors:** Ferdinand Emaniel Brel Got, Gervillien Arnold Malonga, Juthèce Privat Malanda-Kiminou, Maryse Akolbout, Ghislain Loubano-Mvoumbi, Dagene Ebourombi, Merlin Diafouka, Gontran Ondzotto

**Affiliations:** 1 Department of Virology and Molecular Viral Oncology, Faculty of Health Sciences, Marien Ngouabi University, Brazzaville, Republic of Congo; 2 Centre de Traitement Ambulatoire de Brazzaville, Brazzaville, Republic of Congo; 3 Hôpital Général de Dolisie, Dolisie, Republic of Congo; 4 Department of Otolaryngology and Head and Neck Surgery, University Hospital Center, Brazzaville, Republic of Congo

**Keywords:** HIV, epidemiology, viral load, virological failure, Brazzaville, Republic of Congo

## Abstract

**Introduction.** Virological failure is one of the main causes of failing to treat, and better management of HIV infection requires understanding and controlling the factors that contribute to this phenomenon. The main objective was to characterize the patients of the active file of the Brazzaville Outpatient Treatment Center in virological failure to identify predictive factors leading to virological failure.

**Methods.** Conducted between June and December 2020, this was a cross-sectional study. Patients enrolled were HIV-1-infected patients from the Brazzaville Outpatient Treatment Center receiving a potent combination therapy for at least 6 months but experiencing virological failure. Viral load was measured using the automated Abbott Real-time HIV-1 m2000rt System. Sociodemographic and clinical data were collected from a computerized patient record software called Santia. For the identification of the independent predictors of virological failure, statistical analysis was performed.

**Results.** A total of 109 patients with virological failure were recruited. The median age of the patients was 45 years (interquartile range: 37–52 years) and women were more represented (74%). More than half of the patients had World Health Organization stage IV HIV and the median duration of antiretroviral treatment was 96 months. The most followed treatment regimen was AZT+3TC+EFV (or nevirapine) with 48%, while the median viral load was 12985 copies ml^−1^.

**Conclusion.** In our study, we did not identify any sociodemographic or clinical variables predictive of virological failure. However, we felt that it would be desirable to carry out a study with temporal follow-up and the possibility of sequencing in order to identify the different circulating genotypes and resistance mutations.

## Data Summary

The authors confirm all supporting data, code and protocols have been provided within the article.

## Introduction

Decades after the discovery of HIV, the advent of highly active antiretroviral therapy (HAART) has greatly improved the life expectancy of people living with HIV, but achieving the UNAIDS 95-95-95 goal, in particular the third target, remains one of the greatest challenges [1]. On a global scale, it has been estimated that out of 38.0 million (31.6–44.5 million) people living with HIV (PLWHIV) at the end of 2019, 25.4 million of PLWHIV were receiving an anti-HIV treatment by the end of the same year and 82% of people on treatment had suppressed viral load (VL) [2,3]. An estimated 42% of the 6.1 million PLWHIV in West and Central Africa knew about their HIV status at the end of 2016. Of these, 83% had access to antiretroviral (ARV) therapy, and of those on treatment, 73% had viral suppression. These data translate into a treatment coverage rate of 35% and a viral suppression rate of 25% among all PLWHIV in the region [1,4]. Treatment failure is a major public health problem, although virological resistance to antiretroviral treatment (ART) is less and less frequent with the introduction of new molecules such as anti-integrases, anti-C-C chemokine receptor type 5 (CCR5) and single-treatment regimens [5,6]. The main objective of HAART is not only to decrease HIV-related morbidity and mortality, with the major challenge being to reduce viraemia and the transmission of HIV by more than 96% [3], but also to decrease and prevent levels of virological failure [7]. Depending on studies and countries, levels of virological failure, with or without resistance mutations in the region of sub-Saharan Africa, in children, range between 13 and 56% and in adults 10 and 41% [8,18]. Thus, several studies have shown that a lot of factors are positively associated with virological treatment failure including poor adherence to treatment [9,10, 12, 13, 19], low baseline CD4 count [8,10, 19, 20], younger age [9,10, 12, 19], longer time on ART [19], male gender [3], advanced World Health Organization (WHO) staging [10], lower current CD4 count [19] and TB/HIV co-infection [7]. In the Republic of Congo, specific studies on therapeutic failure in PLWHIV of all ages and sexes are rare. With a prevalence of 3.4% (2.3–4.4%), the country had 100 000 (78 000–140 000) PLWHIV, of which only 25% (19–35%) were receiving ART [21]. Viral suppression, a key objective for the effectiveness of ART, is unfortunately not achieved in many PLWHIV in the Republic of Congo [22]. It therefore seemed relevant to us to conduct a study on the epidemiological and virological characteristics of PLWHIV under ART for at least 6 months and experiencing virological failure in Brazzaville (Republic of Congo).

## Methods

### Samples

We conducted a cross-sectional study from June to December 2020 on HIV-1-infected patients followed at the CTA of Brazzaville and receiving a potent combination of antiretroviral therapy (cART) for at least 6 months with two consecutive documented VL test results. We defined it as virological failure, a virological rebound after previous suppression of VL or two consecutive VLs over 1000 copies ml^−1^ after 6 months of treatment. At the time of patient registration, the therapeutic guidelines at the CTA were those of the 2016 WHO recommendations, consisting of a dual-nucleoside or nucleotide reverse transcriptase inhibitor (NRTI) plus a non-nucleotide reverse transcriptase inhibitor (NNRTI) as the preferred first-line regimen, while the ritonavir-boosted protease inhibitors were used as a second-line regimen. All patients included in this study were previously diagnosed with HIV-1 according to the national testing algorithm, which consists of three rapid assays, and they were all ART-experienced.

Virological failure as defined in this study was our outcome variable. Sociodemographic characteristics such as age, gender, educational level, marital status, residence (the distance from home to the CTA), profession and clinical characteristics such as WHO clinical stage at initiation, therapeutic line, regimen and duration on ART were the independent variables of the study and were collected from Santia^®^ software (CTA patient database), which is updated after each patient visit. In addition to collecting sociodemographic and clinical characteristics for each patient, about 5 ml of total blood on EDTA was collected for each patient.

### Molecular analysis

HIV RNA was extracted using the Nucli-SENSEasyQ technique, and plasma VL was quantified according to the manufacturer’s instructions with the Abbott^®^ Real Time HIV-1 m2000rt quantitative assay (*m2000 0.6 ml HIV-1 RNA*, *version 2.0*, *m2000rt*; Abbott Laboratories, Chicago, IL), which had a detection threshold of 40 copies ml^−1^.

### Statistical analysis

Statistical analyses were performed with R version 3.4.4 and GraphPad Prism 6 (GraphPad, San Diego, CA, USA) software. Quantitative variables were expressed as the median and interquartile range (IQR), whereas categorical variables were demonstrated with number and percentage. The unpaired t-test and Kruskal–Wallis analysis were used to compare continuous variables, and Chi^2^ or Fischer exact tests were used for categorical variables.

## Results

### Sociodemographic characteristics

A total of 109 HIV-1-infected and ART-experienced patients were included in the study. Women represented three-quarters of the total workforce (81 versus 28). The median age of patients was 45 years (IQR: 37–52). According to marital status, 30 (27.5%) were single, 29 (26.6%) were divorced and 11 (10%) were widowed, while the common-law union were 39 (35.8%). More than half of patients (*n*=72; 66%) had secondary education followed by primary education (*n*=22; 20.1%). Almost 90% (*n*=97) of HIV patients had their residence near their therapeutic care centre. Note that, 38.5% (*n*=42) of patients were unemployed (Table 1).

**Table 1.** Sociodemographic and clinical characteristics of the study population

Characteristics	N (109)	%
Gender		
Female	81	74.0
Male	28	26.0
Age (years)		
Median age (IQR)	45 (37–52)	
Marital status		
Single	30	27.5
Common-law union	39	35.8
Divorced	29	26.6
Widower	11	10.0
Education		
No formal education	4	3.6
Primary	22	20.1
Secondary	72	66.0
University	11	10.0
Residence (Proximity)		
Yes	97	89.0
No	12	11.0
Profession		
Unemployed	42	38.5
Daily labourer	8	7.3
Self-employed	33	30.2
Employed	26	23.8
WHO clinical stage at the initiation of ART treatment		
I	15	13.7
II	7	6.4
III	25	22.9
IV	62	56.8
Therapeutic line		
First line	108	99.0
Second line	1	0.9
Duration on ART (months)		
6	15	13.7
7–36	70	64.2
37–60	24	22.0
ART regimen		
ABC+3TC+EFV (or NVP)	4	3.6
AZT+3TC+EFV (or NVP)	52	47.7
AZT+3TC+LPV/r	1	0.9
EFV+FTC+ TDF (or 3TC)	27	24.7
D4T+3TC+NVP (or EFV)	23	21.1
FTC+TDF+ NVP	2	1.8
Viral load (copies ml^−1^)		
Median VL (IQR)	12 985 (4125–72 844)	

ABC, abacavir; AZT, zidovudine, retrovir; D4T, stavudineEFV, efavirenz; FTC, emtricitabine; LPV/r, lopinavir/ritonavir; NVP, nevirapine; 3TC, lamivudine, epivir; TDF, tenofovir disoproxil fumarate;

### Clinical data

Slightly over half of patients (*n*=62; 56.8%) were in stage IV of the WHO classification when they began their treatment initiation, with almost all patients in first-line therapy (Table 1). During our study, 15 patients (13.7%) had been on ART for less than 6 months, 70 patients (64.2%) had between 6 and 36 months of ART, and 24 patients (22%) were between 36 and 60 months of ARV treatment. The first three most followed treatment regimens were: AZT+3TC+(EFV or NVP) (*n*=52; 47.7%), EFV+FTC+(TDF or 3TC) (*n*=27; 24.7%) and D4T+3TC+(NVP or EFV) (*n*=23; 21.1%), respectively (Table 1).

### Virological data

In this study, the lowest VL was 1064 copies ml^−1^ and the highest was 500 733 copies ml^−1^. The median VL was 129 85 copies per ml (Table 1). Our cohort in virological failure was not tested for resistance (genotyping), but we made a comparison between patients in virological failure with a VL between 1000 and 10 000 copies ml^−1^, on the one hand, and patients having a VL between 10 000 and 100 000 copies ml^−1^, on the other hand. However, no results were statistically significant (Tables2  3).

**Table 2.** Comparison of sociodemographic characteristics between HIV patients with virological failure with VL between 1000 and 10 000 copies ml^−1^ and HIV patients with virological failure with VL between 10 000 and 100 000 copies ml^−1^

Variables	*N*=109 (%)	VL<10e4 (%)	VL>10e4 (%)	*P*-value
*Sex*				
Female	81 (74)	33 (71.7)	48 (73.1)	0.66
Male	28 (26)	13 (28.2)	15 (23.8)	
*Age (years*)				
<43	45 (41.2)	19 (41.0)	26 (41.2)	1
>43	64 (58.7)	27 (59.0)	37 (58.7)	
*Residence (proximity*)				
Yes	97 (89)	42 (91.3)	55 (87.3)	0.55
No	12 (11)	4 (8.7)	8 (12.7)	
*Education*				
No formal	4 (3.6)	2 (4.0)	2 (4.0)	0.95
Primary	22 (20.1)	10 (22.0)	12 (19.0)	
Secondary	72 (66.0)	29 (63.0)	43 (68.0)	
University	11 (10.1)	5 (11.0)	6 (9.0)	
*Marital status*				
Single	30 (27.5)	13 (28.2)	17 (27.0)	0.67
Common-law	39 (35.8)	19 (41.3)	20 (32.0)	
Divorced	29 (26.6)	10 (21.7)	19 (30.0)	
Widower	11 (10.0)	4 (8.7)	7 (11.0)	
*Profession*				
Unemployed	42 (38.5)	16 (34.7)	26 (41.0)	0.24
Daily labourer	8 (7.3)	5 (10.8)	3 (5.0)	
Self-employed	33 (30.2)	11 (23.9)	22 (35.0)	
Employed	26 (23.8)	14 (30.4)	12 (19.0)	

**Table 3.** Comparison of clinical data between HIV patients with virological failure with VL between 1000 and 10 000 copies ml^−1^ and HIV patients with virological failure with VL between 10 000 and 100 000 copies ml^−1^

Variables	*N*=109 (%)	VL<10e4 (%)	VL>10e4 (%)	*P*-value
*WHO clinical stage at the initiation of ART treatment*	*	*	*	
I	15 (13.7)	7 (15.0)	8 (13.0)	
II	7 (6.4)	2 (4.0)	5 (8.0)	
III	25 (22.9)	10 (22.0)	15 (24.0)	0.89
IV	62 (56.8)	27 (59.0)	35 (55.0)	
*Duration on ART (months)*	*	*	*	*
6	15 (13.7)	8 (17.0)	7 (11.0)	
6–36	70 (64.2)	29 (63.0)	41 (65.0)	0.63
36–60	24 (22.0)	9 (20.0)	15 (24.0)	
*ART Regimen*	*	*	*	*
ABC+3TC+EFV (or NVP)	4 (3.6)	2 (4.3)	2 (3.1)	
AZT+3TC+EFV (or NVP)	52 (47.7)	26 (56.5)	26 (41.2)	
AZT+3TC+LPV/r	1 (0.9)	0	1 (1.58)	
EFV+FTC+ TDF (or 3TC)	27 (24.7)	10 (21.7)	17 (27.0)	1
D4T+3TC+NVP (or EFV)	23 (21.1)	7 (15.2)	16 (25.4)	
FTC+TDF+NVP	2 (1.8)	1 (2.1)	1 (1.5)	

ABCabacavirAZTzidovudine, retrovirD4TstavudineEFVefavirenzFTCemtricitabineLPV/rlopinavir/ritonavirNVPnevirapine3TClamivudine, epivirTDFtenofovir disoproxil fumarate

## Discussion

Virological failure is one of the major constraints to achieving UNAIDS goal of zero HIV infection by 2030. It is therefore crucial to understand, and even master, the various mechanisms that lead to virological failure in order to take appropriate measures to improve care for PLWHIV. This study aimed to characterize HIV-1-infected and ART-experienced patients of the active file of the CTA of Brazzaville who were facing virological failure after ART initiation for at least 6 months. Unlike other studies that have identified being male in gender [3], younger age [9,10, 12, 19], low current body mass index (BMI) [3], poor adherence to treatment [9,10, 12, 13, 19], advanced WHO staging [10], low baseline and low current CD4 count [8,10, 19, 20], longer duration on ART [19] and TB/HIV co-infection as factors positively associated with virological treatment failure, some are subject of consensus and others not. Our study found no statistically significant relationship between sociodemographic or clinical variables and virological treatment failure. This could be explained by the fact that we conducted a cross-sectional study with a single sample per patient at a given time, and we believe that a follow-up over time would certainly have revealed more arguments linked to virological failure. The other possible reason might be the fact that in these studies, they used a higher sample size compared to our study; and it is clear that as the size of the sample increases, so does the probability of getting a higher number of events. Also, compared to these other studies, we can also mention the difference in inclusion criteria based on the advanced WHO clinical stage (stage IV or late-stage III in other cases) or low baseline CD4 count.

However, one of the main factors influencing virological failure is therapeutic compliance, and it is a common agreement that when the level of adherence is less than 95%, patients are at risk of developing virological failure due to the fact that poor adherent patients often have low basal CD4 cell count, which provides a favourable environment for the virus to replicate [10,23]. Although we have no data on patient’s adherence, we can nevertheless note some facts influencing patient’s adherence: nearly 40% of patients were unemployed and so might neglect treatment because of difficulties in eating, which would demotivate them to stick to taking their medication. El-Khatib *et al.* in South Africa reported the same observation; he noted that, concerning self-reasons for not taking any of their pills, some patients stated that they do not want to take the pills on an empty stomach, as they do not have anything to eat [24]. Another factor leading to poor adherence is the duration on ART; in this study, 64% of patients were experiencing ART for a period of 6–36 months, which could lead to a loss of interest in the treatment over time, especially when taking several tablets, a day. One of the other important factors linked to poor adherence that we can also point to is the untimely rupture of ARV molecules, as it is clear that in the presence of low plasma concentrations of ARV, the virus replicates and accumulates resistance mutations, leading to treatment failure [3,7]. It is important to note that during the period of our study, the country experienced periods of untimely interruptions in the supply of ARVs for political and economic reasons, in particular non-payment of invoices to central purchasing agencies. We would also have liked to take this study to the molecular characterization of HIV strains, which would have allowed us to identify resistance, given to the wide genetic diversity of HIV-1 that had been reported in the country (such as the global analysis of the study by Ferdinand *et al.* in 2021 showed that the HIV-1 CFR02_AG was the predominant subtype with the prevalence of 22%, the most frequent mutations conferring resistance to NRTIs and NNRTIs was, respectively, the M184V/I with 37% and the K103N with 26% while, the major protease inhibitor (PI) mutations responsible for drug resistance (I47V, L76V) counted for less than 5%) [25,26] because high viral loads may be associated with multidrug resistance rather than poor adherence. Also, because it is important to assess the potential risk of transmission of ARV-resistant virus strains in developing countries, then monitoring resistance mutations in each country is part of achieving the UNAIDS 95-95-95. Limitations of this study are essentially linked to the method of data collection, which did not allow us to obtain information on certain variables, both sociodemographic and clinical, that could be studied, such as BMI, adherence, co-infection notion and nutritional status. We also deplored the lack of data on CD4 T-cell counts, due to the fact that the laboratory was short of reagents at the time of our study, and it is well known that the lower the CD4 count, the more active the viral replication. This prevented us from assessing the immunological status of patients and studying the links with virological treatment failure.

## Conclusion

The present study revealed that none of our sociodemographic or clinical variables is a determining factor in virological treatment failure. Although contrary to what has been demonstrated in other studies, and despite the limitations of our study, we would like to have seen a longer follow-up period for patients in virological failure, with the possibility of carrying out HIV resistance genotyping in these patients in order to identify circulating genotypes.

## Abbreviations

ART: antiretroviral treatment
ARV: antiretroviral
cART: combination of antiretroviral therapy
CCR5: C-C chemokine receptor type 5
HAART: highly active antiretroviral therapy
IQR: interquartile range
NNRTI: ucleotide reverse transcriptase inhibitor
NRTI: nucleotide reverse transcriptase inhibitor
PI: protease inhibitor
PLWHIV: people living with HIV
UNAIDS: Joint United Nations Programme on HIV/AIDS
VL: viral load
WHO: World Health Organization
